# Rhinocerebral Mucormycosis Associated With Anterior Skull Base Actinomyces Osteomyelitis in a Pediatric Patient With Type 1 Diabetes

**DOI:** 10.7759/cureus.24311

**Published:** 2022-04-20

**Authors:** William Conley, Ronald E Cox, Thomas Robey

**Affiliations:** 1 Department of Otolaryngology-Head and Neck Surgery, Medical College of Wisconsin, Milwaukee, USA

**Keywords:** diabetes mellitus type i, pediatric infectious disease, functional endoscopic sinus surgery, actinomycetes, rhinocerebral mucormycosis

## Abstract

Rhino-orbital-cerebral mucormycosis (ROCM) is a fulminant, often fatal, angioinvasive fungal infection commonly transmitted through inhalation of fungal spores and traumatic inoculation. While the literature has documented rare cases of infection in immunocompetent patients, the vast majority of case fatalities are noted in immunosuppressed populations. Common predisposing factors to infection include immunosuppressive therapies, hematologic malignancies, and most notably, uncontrolled diabetes. Actinomycosis is a subacute to chronic bacterial infection stemming from non-spore-forming anaerobic/microaerophilic bacteria of the genus Actinomyces. Infection with Actinomyces species has been documented across numerous anatomical sites; however, literature on concurrent infection with ROCM in pediatric patients is sparse. We document a case of a 17-year-old male with uncontrolled type 1 diabetes who presented to the emergency department with combined ROCM and actinomycotic infection of his anterior skull base.

## Introduction

Mucormycosis is a rapidly progressive, opportunistic infection arising from numerous mycotic species in the Mucorales order [1]. This disease is commonly transmitted by inhalation of fungal spores into the nasopharynx, paranasal sinuses, and lungs. There are also incidences in the literature reporting direct inoculation secondary to cutaneous injury [2]. Mucormycotic infections are commonly classified by the origin of the anatomic location of infection. The rhinocerebral type is well documented due to its life-threatening complications, secondary to fungal invasion of the brain. Within the subdivisions of the rhinocerebral classification, rhino-orbital-cerebral mucormycosis (ROCM) is thought to have the highest incidence of mortality [3].

The primary organism documented in 90% of ROCM cases is the aseptate filamentous fungus, *Rhizopus oryzae* [4]. This saprotrophic fungus has a proclivity to promote angiogenesis and rapid proliferation in its host species. ROCM most commonly presents in immunocompromised patient populations, such as those with a history of chronic steroid use, underlying malignancy, and uncontrolled diabetes. Patients experiencing diabetic ketoacidosis (DKA) have a higher chance of developing ROCM [5]. Not only does excess glucose promote fungal growth, but the acidotic environment created in DKA also allows for excessive free iron accumulation [6]. These conditions foster a conducive setting for rapid fungal proliferation. Commonly noted systematic signs of infection include fever and malaise, while localized ophthalmic symptoms reported include proptosis, chemosis, periorbital edema, and ophthalmoplegia [4]. 

Actinomycosis is a rare suppurative bacterial infection first documented in human cases over a century ago [7]. Actinomyces species are commonly found in the human microbiota, particularly in the oral cavity, digestive system, and genital tract. While infection with Actinomyces is most commonly from an odontogenic spread in the cervicofacial region (60%), actinomycotic infiltration of the nasal cavity, paranasal sinuses, and skull base is exceptionally rare [7,8]. Pathogenic strains of Actinomyces have been linked to granulomatous disease, tissue fibrosis, and sinus tract formation [1,9]. Like ROCM, actinomycosis is frequently documented in patients with adaptive and innate immunodeficiencies. 

There are only a handful of cases of skull base actinomycosis reported and two cases of combined ROCM and maxillary actinomycosis [1,9]. We report an exceedingly rare case of a 17-year-old male patient, with uncontrolled type 1 diabetes mellitus, who presented with ROCM and actinomycosis of his paranasal sinuses and anterior skull base.

## Case presentation

A 17-year-old male presented to the emergency department at Children’s Wisconsin with complaints of right eye pain, periorbital swelling, and blurry vision. The patient had a prior medical history of uncontrolled type 1 diabetes, having been hospitalized 14 times since 2014 for DKA. The patient reported his glucose levels had ranged in the 300-400s for several days preceding this emergency room visit. He denied symptoms of fever, headache, or facial pain. The patient was afebrile with age-appropriate vitals. On physical examination, there was significant swelling of the right orbit with mild proptosis. He had limited, painful extraocular motion, injected conjunctiva, and clear drainage from the right puncta. His pupils were reactive; however, his visual acuity was decreased. The remainder of the physical exam was unremarkable. 

**Figure 1:**
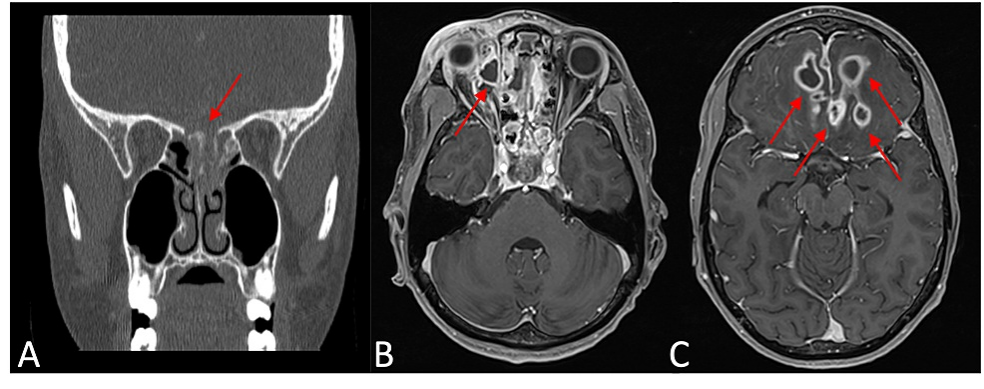
Preoperative CT and MRI images obtained after initial presentation. (A) Coronal CT showing dehiscence of the cribriform plate and opacification of left posterior ethmoid cells; (B) T1 axial MRI showing subperiosteal abscess in right medial orbit; and (C) T1 axial MRI showing bilateral frontal lobe abscesses.

Laboratory findings revealed an elevated glucose level of 360 mg/dL, venous pH of 7.416, elevated venous bicarbonate of 29.2 meq/L, elevated white blood cell count of 15.7 × 10^9^/L, and an erythrocyte sedimentation rate over 100 mm/h. An orbital computed tomography (CT) was obtained demonstrating a rim-enhancing fluid collection in the medial right orbit adjacent to the right ethmoid air cells. Complete opacification was noted in the bilateral frontal, ethmoid, and sphenoid sinuses. Maxillary sinuses contained minimal mucosal thickening. The scan revealed dehiscence of the cribriform plate, and a fluid collection was seen in the anterior cranial fossa. Most notably, multiple rim-enhancing fluid collections were seen throughout the parenchyma of the bilateral frontal lobes. A brain magnetic resonance imaging (MRI) study was obtained to further elucidate the CT findings (Figure 1). Sinus and anterior cranial fossa findings were confirmed and a subperiosteal abscess was noted in the right medial orbit. Four fluid collections were found in the frontal lobes, three located in the left parenchyma and one within the right. 

Empiric IV clindamycin (500 mg every eight hours) and IV ceftriaxone (2 g every 12 hours) were started on admission. The pediatric otolaryngology team was consulted, and the patient was taken to the operating room one day after admission. The patient underwent a right frontal sinusotomy, right maxillary antrostomy, right total ethmoidectomy, and bilateral sphenoidotomy. The oculoplastic team performed a right medial orbitotomy for drainage of his subperiosteal abscess. Purulence was noted throughout, and multiple cultures were taken. It was also noted that the right cribriform plate was completely eroded and contained pockets of purulence extending intracranially. An intraoperative pediatric neurosurgical consultation was obtained, and the decision was made to leave the intracranial portion alone. The posterior right middle turbinate was also noted to be necrotic at the skull base; it was removed and sent for biopsy. The adjacent skull base was soft and friable with obvious purulence and areas of yellow phlegmon-type debris. This area was cultured and had multiple samples taken for biopsy. Intraoperative samples of the left sphenoid sinus came back positive for fungal elements so both sides of the nose were extensively irrigated with amphotericin B. Results of cultures and biopsy samples taken intraoperatively are listed in Table 1 and Table 2, respectively. Most notably the left sphenoid sinus cultured *Rhizopus oryzae*, and cultures of the anterior skull base eventually grew out Actinomyces.

**Table 1:** Results of intraoperative cultures taken from various areas of the paranasal sinuses and skull base.

Culture area	Findings
Right anterior skull base	*Staphylococcus epidermidis*,Corynebacterium,* Streptococcus mutans*,Actinomyces
Right frontal sinus	Staphylococcus epidermidis
Right posterior ethmoid	*Staphylococcus epidermidis*,Corynebacterium, Lactobacillus
Left sphenoid	*Staphylococcus epidermidis*,Corynebacterium,* Rhizopus oryzae*

**Table 2:** Results of biopsies taken from various areas of the paranasal sinuses and skull base.

Biopsy Samples	Findings
Left posterior sinus contents	Fibrous tissue and bone with active suppurative inflammation and necrosis, involved by fungal hyphae
Right middle turbinate	Sinonasal tissue with active chronic inflammation, negative for definite fungal hyphae
Right nasal contents	Sinonasal tissue with mild chronic inflammation, negative for fungal hyphae
Right skull base	Inflamed fibrocollagenous tissue and bone with active chronic inflammation and necrosis, involved by fungal hyphae

The patient’s hospital course lasted 12 weeks and required extensive anti-fungal and antibacterial treatment administered through a right basilic vein peripherally inserted central catheter (PICC) line. He was started on anti-fungal therapy including liposomal amphotericin B (187 mg IV daily) and posaconazole (250 mg IV daily). Both medications were administered throughout the entire hospitalization. Six retrobulbar amphotericin B injections were also conducted. Prior to discharge, the patient’s PICC line was transitioned to a right internal jugular vein port for ease of use at an infusion clinic. The IV amphotericin B was continued outpatient for an additional 13 weeks, based upon multidisciplinary recommendations. Additionally, posaconazole was changed from IV to an oral formulation with a dosage of 300 mg twice daily. 

The primary antimicrobials used were metronidazole and ceftriaxone. Additionally, clindamycin was started empirically on presentation for methicillin-resistant *Staphylococcus aureus* (MRSA) coverage. However, after one day of treatment, the infectious disease department recommended discontinuing the clindamycin for IV vancomycin. The vancomycin was continued for one week and then subsequently transitioned to linezolid secondary to concerns of renal toxicity in the setting of a rising creatinine. The patient also received ophthalmic erythromycin ointment to the right eye daily. Table 3 below denotes the duration and dosage of each antibiotic treatment administered. 

**Table 3:** Duration and dosage of antibiotic treatments

Antibiotic	Dosage	Duration of treatment
Clindamycin	500 mg IV every 8 hours	1 day
Metronidazole	375 mg IV every 6 hours	4 weeks
Ceftriaxone	2 g IV every 12 hours	4 weeks
Vancomycin	600 mg IV every 6 hours	1 week
Linezolid	370 mg PO every 12 hours	3 days

In addition to antimicrobial treatment, the patient received hyperbaric oxygen therapy five times weekly for a duration of two months. Serial MRI showed a decreasing size of the frontal lobe abscesses and epidural empyema. Persistent inflammation remained along the right medial orbit involving the superior oblique and medial rectus muscles. Diffuse paranasal sinus disease has also remained; however, there are no signs of new fluid collection.

## Discussion

We report a rare pediatric case of combined ROCM and skull base actinomycosis in a diabetic patient which required urgent surgical debridement, aggressive medical correction of his blood glucose levels as well as hyperbaric oxygen therapy. Mucormycosis is an angioinvasive fungal infection caused by more than 30 pathogenic species within the Mucorales order; the two most common being Rhizopus and Mucor. Mucormycosis can be classified based on anatomic location of fungal invasion with rhino-orbital-cerebral (44-49%) being the most common [1]. ROCM frequently presents with complaints of fever, headache, sinus pain, and retro-orbital pain. Infection typically starts within the nasal mucosa and involves the sinuses, orbit, and skull base, and can extend into the cranial cavity. Patients with cancer, organ transplantation, and chronic steroid use all have an increased risk of ROCM. However, the highest incidence of ROCM is seen in diabetics; a report from Reed et al. in 2008 found that 83% of patients with ROCM had underlying diabetes [10]. Moreover, the hyperglycemic and acidic environments decrease mobilization, chemotaxis, and phagocytic activity of polymorphonuclear leukocytes (PMN). The amount of PMN apoptosis is escalated by inhibition of glucose-6-phosphate dehydrogenase (G6PD), necessary for cell survival. Levels of serum-free iron, needed for fungal proliferation, increase in the lower pH blood of diabetics. Patients experiencing DKA are more likely to develop ROCM caused by *Rhizopus oryzae* as the organism thrives with a ketoreductase system, high levels of iron, hyperglycemia, and low oxygen conditions [11].

Actinomycosis is an uncommon subacute to chronic bacterial infection caused by members of the *Actinomyces* spp., which are filamentous anaerobic Gram-positive bacilli [7]. Normally found throughout the alimentary canal and genital tract, infection also typically affects immunosuppressed individuals. Infection proliferates through mucosa injury or breakdown, but it is the presence of devitalized tissue that allows the infection to spread into deep structures. *Actinomyces* species are rare to cause infection by themselves, as they have relatively low pathogenicity within hosts. Actinomycosis involves multiple members within the Actinomyces, Propionibacterium, Bifidobacterium, and other genera. A single infection can contain between one and 10 bacterial species in addition to the disease-causing actinomyces species. Companion bacteria assist Actinomyces in forming toxins or enzymes, furthering tissue destruction and immune suppression. Contiguous spread of the infection is common, with the potential for the formation of draining fistulas or hematogenous dissemination to distant sites. Once the infection has taken root, an intense suppurative and granulomatous immune response is triggered with possible fibrosis results. The anatomic location of actinomycosis varies but the cervicofacial (50%) region is most common. Rarely do these infections involve the central nervous system and bone [12]. Actinomyces osteomyelitis is a rare presentation of actinomycosis, but the involvement of the skull base has only been reported in a handful of cases [13,14].

Our patient’s presentation uniquely illustrates a rare combination of microbial diseases. The precipitating event of this patient’s disease presentation was his uncontrolled diabetes mellitus. A challenging social situation also precluded adequate diabetes management. Considering the patient hosted an ideal environment for *Rhizopus oryzae* and ubiquitous airborne fungal spores, it is possible that fungal spread began weeks before presentation, resulting in this rapid and uncontrolled progression. CT and MRI findings in similar acute ROCM cases show bone destruction of sinus walls, skull base erosion, and intracranial and intraorbital extension of inflammation, as well as abscess formation in the cerebral parenchyma [15]. Additionally, most patients with cases of ROCM report symptoms similar to acute sinusitis including facial pain, headache, and rhinorrhea [16]. Our patient had an absence of the typical sinus symptoms seen in other cases. We hypothesize that in addition to damage caused by infection, his longstanding and undermanaged hyperglycemia led to injury of peripheral branches of the trigeminal nerve throughout the paranasal sinuses leading to sinus paresthesia.

As mentioned above, there are very few reports of actinomycosis invasion of the skull base [8]. In the cases reviewed, patients had an overwhelming history of dental extraction or chronic rhinosinusitis which eventually resulted in osteomyelitis of the skull base [13,17,18]. Our patient did not have a history of dental procedures before presentation, so it is unclear as to the origin of the Actinomyces infection. It is possible that he had an underlying dental disease that will require more aggressive treatment after his discharge. Although combined ROCM and actinomycosis cases have been reported in the literature previously, this is the first case report where the Actinomyces infection involved the skull base. 

Our patient’s treatment plan included the mainstay of ROCM treatment including surgical debridement, antifungals (amphotericin B and posaconazole), and hyperbaric oxygen therapy. Actinomycosis treatment also consists of surgical debridement and B-lactam antibiotics. The duration of antibiotic and antifungal treatment varies between eight to 12 weeks and four to six weeks, respectively. Our treatment plan combined surgical intervention with aggressive bacterial and fungal medical treatment. Two months of daily hyperbaric oxygen therapy was used as fungal suppressive therapy for cerebral abscesses. A supplementary challenge with this patient was the tight management of his glucose levels which required hourly checks and numerous adjustments of sliding scale insulin.

Recognition of this combined disease can be challenging if there is a lack of physical signs or symptoms. Aggressive surgical therapy based on imaging results is necessary for prompt treatment and allows for culture and biopsy-directed results to optimize medical therapy. These actions are necessary to prevent further intracranial spread and possible death. It is important for otolaryngologists to have knowledge of these disease processes and management options to prevent significant morbidity and mortality. 

## Conclusions

This case report highlights that pediatric patients presenting with uncontrolled diabetes mellitus have a significant risk for opportunistic infections. Early detection and management of comorbid factors undoubtedly play a significant role in mitigating disease. The triad of DKA, mucormycosis, and actinomycosis at presentation intensify the clinical complexity of care. High clinical suspicion of infection and invasive sampling procedures are vital in establishing a timely diagnosis and treatment plan.
